# The impacts of okra consumption on the nutritional status of pregnant women, west Ethiopia

**DOI:** 10.1002/fsn3.3512

**Published:** 2023-06-16

**Authors:** Efrem Negash Kushi, Tefera Belachew, Dessalegn Tamiru

**Affiliations:** ^1^ College of Health and Medical Science Mettu University Mettu Ethiopia; ^2^ Departments of Nutrition and Dietetics Jimma University Jimma Ethiopia

**Keywords:** mid‐upper arm circumference, okra, pregnant women, west Ethiopia

## Abstract

Food‐insecurity and poor‐quality diets remain a challenge for pregnant women. Consumption of okra has a great impact on improving the nutritional status of pregnant women. Okra plays a critical role in the prevention of malnutrition among pregnant women living in resource‐limited settings. The evidence is scarce on the impacts of okra on mid‐upper arm circumference (MUAC) measurement of pregnant women. A community‐based cross‐sectional study was employed among randomly selected 224 pregnant women from June 1 to July 30, 2020. An interviewer‐administered questionnaire was used. The MUAC was measured by using an adult MUAC measuring tape. Data were entered into Epi‐data version 3.1 and exported to SPSS version 25 for analysis by linear regression. The statistical significance of variables was declared at a *p‐*value of .05, and unstandardized beta (*β*) coefficients along with a 95% confidence interval were computed. The proportion of pregnant women with low MUAC (≤22 cm) was 42.4%. In the multivariable linear regression model, hemoglobin concentration (*β* = 0.346, [95% CI: 0.153, 0.539], *p* = .001), food insecurity (*β* = −0.887, (95% CI: −1.441, −0.334), *p* = .002), daily consumption of okra (*β* = 1.269, [95% CI: 0.583, 1.956], *p* ≤ .001), and women working in NGO (*β* = 0.443, [95% CI: 0.256, 0.630], *p* ≤ .001) were significant variables. The prevalence of malnutrition among pregnant women (MUAC ≤ 22 cm) was 42.4%. Therefore, behavioral change communication interventions to promote okra consumption on regular basis were recommended.

## Graphical abstract

Proportion of pregnant women with low MUAC (≤22 cm) was 42.4%. Consumption of okra has a significant impact on improving the nutritional status of pregnant women.

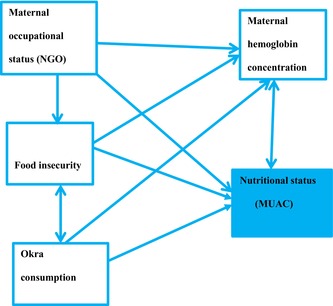

## 1 INTRODUCTION

Adequate nutrition is a fundamental cornerstone of women's health (Nguyen et al., 2014). A woman whose diet is adequate before pregnancy reduces the risk of birth‐related complications (Williamson, 2006). But globally, the problems of under as well as overnutrition are common in the female population (World Health Organization, 2016). Likewise, food insecurity and poor‐quality diets remain a challenge (Maina et al., 2017). Thus, the intake of a diverse diet is a cost‐effective strategy to overcome this problem (Bhandari et al., 2016). Globally, women in rural areas who consumed a diversified diet had better dietary diversity scores (Shashikantha et al., 2016).

Malnutrition poses a variety of threats to pregnant women and efforts to promote the consumption of local nutrient‐rich foods are needed (Elder & Ransom, 2003). Moreover, Sub‐Saharan Africa is home to some of the most nutritionally insecure people in the world (Fanzo, 2012). Besides this, around 85% of households in rural areas use a diversity of wild edible plants to satisfy the diversity of their food (Aryal et al., 2018; Misra et al., 2008; Namrata et al., 2011). Wild edible plants' nutritional content is higher than those of domesticated field crops (Borelli et al., 2020; Carvalho & Barata, 2016; Grivetti & Ogle, 2000). Likewise, wild edible plants like okra have great cultural importance to the rural population in resource‐limited settings (Misra et al., 2008). Edible plants such as okra are the staple food, supplements for a nutritionally balanced diet, and also a source of income for poor communities in different parts of the world (FAO, 2006; Shrestha & Dhillion, 2006).

Edible plants like okra have a critical role in ensuring food security and prevention of malnutrition, particularly for pregnant women (Salvi & Katewa, 2014). Okra is important during pregnancy and lactation (Humphry et al., 1993; Villard & Bates, 1987). Okra is an important vegetable crop that is used to have various health benefits such as antidiabetic properties (Dubey & Mishra, 2017). It is a very good source of dietary fiber, different minerals, and vitamins, especially folate, B1, and B6. It is also rich in antioxidants (flavonoids) and is rich in essential fatty acids (linoleic acid), which are very important for human nutrition (Badrie, 2016; Durazzo et al., 2018). Moreover, okra is an excellent source of folate, which promotes healthy pregnancy. This is used to build and maintain new cells, which are very important for maintaining appropriate fetal brain development as well as preventing birth defects (spina bifida) (Okra H, n.d.).

Evidence showed that food fortification with okra seed flour resulted in an increase in different nutrients which improves the nutritional status of pregnant women (Adetuyi & Adelabu, 2011). Furthermore, okra seeds were the antifatigue part of okra pods while polyphenols and flavonoids were active constituents that promote the antioxidant role of the plant (Dantas et al., 2021; Xia et al., 2015). Furthermore, okra is used for anti‐inflammatory, immunomodulatory, antibacterial, anticancer, antidiabetic, organ‐protective, and neuropharmacological activities (Islam, 2019; Panighel et al., 2022; Sipahi et al., 2022). In line with this, animal experiments indicated that okra suppresses insulin resistance, thereby improving the blood glucose level of gestational diabetic mellitus (Tian et al., 2015). Moreover, clinical trials indicated that supplementation of dried okra extract could significantly decrease energy intake and carbohydrate consumption in diabetic nephropathy patients (Nikpayam et al., 2022). For this reason, okra is considered to be an economically affordable vegetable crop with various potential health benefits and improves the nutritional status of pregnant women (Elkhalifa et al., 2021).

However, the dietary consumption of wild edibles has decreased as the dietary intake pattern of people worldwide is changing from a traditional diet to a modern diet (highly processed foods) (Kuhnlein & Receveur, 1996). These lifestyle changes lead to the double burden of malnutrition in low‐income countries (Popkin et al., 2020).

The use of wild edible plants as food sources is common in Ethiopia (Getahun, 1974). The most common traditional food source for indigenous communities of western Ethiopia is a plant locally named “*Kenkase*.” This plant was commonly named “Okra” (Huang et al., 2007). It is an important vegetable crop cultivated in tropical and subtropical regions of the world (Singh et al., 2014). Okra plays an important role in the human diet, especially for pregnant women (Roy et al., 2014). However, most diets in developing countries lacks this plant (Small, 2006). Okra is a cost‐effective and economically affordable natural source that reduces malnutrition in resource‐limited settings (Elkhalifa et al., 2021).

Studies showed that the consumption of wild edible plants in Ethiopia is very low covering only 5% of the region in the country's districts (Hailu & Addis, 2016). Furthermore, evidence is scarce on the impacts of okra consumption on mid‐upper arm circumference (MUAC) measurements (nutritional status) among pregnant women. Therefore, this study aimed to assess the impacts of okra consumption on the MUAC measurements of pregnant women, west Ethiopia.

This calls for further evidence that might have inputs that support sustainable development goals such as ending hunger, achieving food security, improving nutrition among nutritionally vulnerable population groups (pregnant women), and promoting sustainable agriculture. Likewise, the study aimed to recommend how to increase the agricultural productivity of okra with other cultivated crops in the area. Similarly, the finding of this study was used to improve the food quality and nutritional status of pregnant women through the promotion of diversified agricultural products. This might have also input that supports sustainable development goals such as ensuring sustainable production as well.

## 2 METHODS AND MATERIALS

### 2.1 Study design

A community‐based cross‐sectional study design was employed from June 1 to July 30, 2020.

### 2.2 Study setting

This study was conducted in the Sherkole and Asossa districts of western Ethiopia. The Asossa Zone is located in the Benishangul Gumuz Regional State, west Ethiopia. The latitude and longitude of the region are 10°04′ N 34°31′ E/10.067° N 34.517° E/10.067; 34.517, with an elevation of 1570 meters above sea level. The indigenous communities in the region are Berta, Gumuz, Shinasha, Maho, and Komo. The regional city is Asossa Town which is 670 Km far away from the capital city of Ethiopia. The total population of the region is 405 and 466 (Benishangul Gumuz regional statics agency report of 2015, unpublished report), and there are a total of 8324 and 30,049 women in the reproductive age group of Berta Communities found in the Sherkole and Asossa districts respectively. The climatic condition of the Asossa zone is tropical (Benishangul Gumuz Regional Health Beruae District Plan of 2019, unpublished work).

### 2.3 Source population

All households with pregnant women of Berta Community who live in the Asossa Zone were source populations.

### 2.4 Study population

All households of pregnant women of the Berta community and first trimester who have been included in the sample of selected kebeles were the study population. Households with at least one pregnant woman were included. For more than one eligible woman in the selected households, the one who was responsible for family care was considered for this study.

#### 2.4.1 Inclusion criteria

Pregnant women of the Berta community who are permanent residents (who live in the study area for a duration of more than or equal to 6 months), in first‐trimester pregnancy, and who use the Okra plant as their food source were included in this study.

#### 2.4.2 Exclusion criteria

Those Berta community women who are critically ill, not willing to give consent, and second and third‐trimester pregnancies were excluded.

### 2.5 Sample size determination

The sample size was calculated by using G*Power 3.0 based on the following assumptions: *Z*‐tests of difference between two independent proportions (*p*1 = .5 and *p*2 = .6), power of 80%, and a margin of error 5% with 95% confidence level. Thus, the sample size, by considering the design effect of 2, was 204 pregnant women. Finally, by considering the 10% nonresponse rate, 224 pregnant women of the Berta community were used.

### 2.6 Sampling procedure

A multistage sampling technique was used. Likewise, kebeles were selected by a simple random sampling method. The identification and registration of women with known pregnancies were performed from each household using the pregnancy screening checklist. The screening checklist consisted of six items with “Yes” or “No” responses. The items were: questions that asked about delivery and breast‐feeding in the last 6 months, delivery in the last 4 weeks, the menstrual period in the last 7 days, abortion, or miscarriage in the last 7 days, sexual abstinence since the last menstrual period, and the current use of contraceptives. In addition to this, the urine of the woman who was suspected as pregnant was tested for confirmation using a WHO rapid test kit (human gesells chaft für biochemical and diagnostics mbh, Wiesbaden, Germany) (Stanback et al., 2008). Finally, eligible first‐trimester pregnant women were selected by a simple random sampling technique.

### 2.7 Data collection tool and procedure

The data were collected using a pretested, interviewer‐administered structured questionnaire.

#### 2.7.1 Mid‐upper arm circumference

It was assessed by using an adult MUAC nonstretchable measuring tape on the left arm relaxes along the body trunk. The reading was taken to the nearest 0.1 cm (Abrhame & Haidar, 2014).

#### 2.7.2 Hemoglobin concentration

This was measured by taking a finger‐prick blood sample from each woman using a HemoCue Hb 301 (HemoCue AB). Then, a prick was made on the tip of the middle finger after the site was cleaned with disinfectant. The first drop of blood was cleaned off while the second drop was collected to fill the microcuvette for measuring hemoglobin concentration. Finally, hemoglobin concentration below the cutoff of 11.0 g/dL in pregnant women was taken for anemia (Murphy, 2011).

#### 2.7.3 Dietary practices

It was evaluated by eight structured questionnaires which had internal consistency (Cronbach's alpha of 0.82). Each question was given one mark if the answers were healthy for dietary practices and also zero scores were given if the responses were unhealthy for dietary practices. Finally, pregnant women were classified to have poor dietary practices if they correctly answered <75% of practice questions and good dietary practices if they correctly answered ≥75% of questions (Mahmood et al., 1997). Finally, a dummy variable was created and coded.

#### 2.7.4 Dietary diversity

It was assessed by a qualitative open 24‐h recall: the enumerator asked a series of standard probing questions to help the women recall all foods and beverages consumed the previous day and night. He/she also probes for the main ingredients in mixed dishes. Specifically, the recall period covers from when the respondent awoke the previous day, through the day and night for 24 h. Each food or beverage that the respondent mentioned can be circled or ticked on a predefined list. Foods not already included on the predefined list can be either classified into an existing predefined food group or recorded in a separate place on the questionnaire and coded later into one of the predefined food groups (FAO, 2016). Finally, dummy variables were created for linear regression analysis.

#### 2.7.5 Knowledge of diversified diet

A total of 12 items were used to measure the knowledge of participants for the use of a diversified diet. The items had internal consistency (Cronbach's alpha of 0.87). The scores of knowledge‐related questions were converted into tertials. Measurements those who had the highest tertial were categorized as good knowledge, while the two lower tertials were combined and labeled as poor knowledge. Finally, for linear regression analysis, dummy variables were created and coded.

#### 2.7.6 Attitude toward diversified diet

Participants' attitude was measured using a 5‐point Likert scale (Cronbach's alpha of 0.89). The final scores of the Likert scale were computed and then converted into tertials. Those who had the highest tertial were categorized as having a positive attitude, while the two lower tertials were combined and labeled as negative attitudes. Finally, dummy variables were created and coded for linear regression analysis.

#### 2.7.7 Household food insecurity

To assess household food insecurity, a series of nine questions were presented to pregnant women. The questions were asked during the past month to assess the experiences of a pregnant woman during a lack of access to food. The “yes” responses were coded one, and the “no” responses were coded zero. The responses were summed to produce an index of household food insecurity. The index was divided into tertials and a dummy variable was created. Participants grouped in the highest tertial were labeled as “food insecure” and coded 1, while the two lower tertials were combined and coded 0 to be labeled as “food secure” (Belachew et al., 2013).

#### 2.7.8 Frequency of okra consumption

It was assessed by pretested interviewer‐administered questionnaires. Dummy variables were created by categorizing participants who consumed okra daily as coded 1, while those who consumed sometimes were coded 0.

### 2.8 Data quality control

Data quality was ensured by training data collectors and supervisors on the purpose of the research, how to use the data collection tools, and the validation of measuring instruments. Furthermore, the English version questionnaires were translated into the native language (Rutanegna) of the respondents by language experts and then back to the English version to check its consistency. Likewise, the collected data were checked out for completeness, accuracy, and clarity. Moreover, a pretest of instruments for data collection was done among 45 pregnant women in the Bambasi district, western Ethiopia. Finally, data cleanup and cross‐checking were also done before the data analysis.

### 2.9 Ethical considerations

Ethical approval was secured from Inistitutional Review Bord of Jimma University, Institute of Health. Likewise, an official letter of cooperation had given to Inistitutional Review Bord of Jimma University, Institute of Health. Moreover, the local authorities were informed about the research through an official letter. In addition to this, adequate information was provided to the respondents about the research and their right to participation, their right to decline participation any time they feel to do so. Pregnant women with health problems based on their verbal complaints were informed to visit the public health facilities nearer to them. The participants of the study were also assured that their treatment and other benefits they gained from the health institutions and other organizations were not influenced by their participation in the study. Likewise, they were asked for their informed written consent to participate in the study. For those women below the age of 18 years, written consent to participate in the study was obtained from their guardians. Finally, pregnant women had been told that all the data taken were not disclosed to other bodies to maintain their confidentiality.

### 2.10 Data processing and analysis

Collected data were entered into Epi‐data version 3.1 and then exported to SPSS version 25 for analysis. Dummy variables were created for categorical variables and coded with participants having attribute 1 while those not having 0 with their respective number of variable categories. The number of dummy variables was computed as (*K* − 1) where *K* is the number of categories. Furthermore, normally distributed continuous variables were presented as means and standard deviations. Linear regression was used and assumptions for binary and multivariable linear regression analyses were checked by: examining the residuals for the linearity, normality, and homoscedasticity assumption. The assumptions were checked with a scatter plot for the P–P plot, and Q‐Q plot, and visually using a histogram. The plots of standardized residuals versus standardized predicted values showed no obvious sign of funneling, and there was no problem of heteroscedasticity.

Furthermore, multicollinearity was checked using a variance inflation factor (VIF) and the value of VIF for all independent variables was less than 10. Moreover, the values of residuals were independent (Durbin–Watson statistics of 1.916), suggesting that there was no problem with autocorrelations of residuals. Finally, outliers were checked by Cook's distance statistic and there were no outliers (the minimum Cook's distance = 0.0001 while the maximum Cook's distance = 0.054). Mean centering for continuous independent variables was created to increase the interpretability of regression coefficients along with dummy‐coded categorical variables. Variables with *p‐*value <.25 were considered for multivariable linear regression analyses to control the possible effects of confounders. Likewise, the statistical significance of variables was declared at a *p‐*value of .05 in the final model while unstandardized beta (*β*) coefficients along with a 95% confidence interval (CI) were computed to measure the strengths of the association between predictors and outcome variables.

## 3 RESULTS

### 3.1 Sociodemographic characteristics of the respondents

A total of 224 pregnant women participated in the study with a response rate of 100%. The mean (SD±) age of respondents was 25.29 (5.63) years. Almost more than half (57.1%) of the respondents were unable to read and write. Likewise, the majority (96.4%) of them were married in terms of their marital status. Regarding their religion, almost all (97.8%) and 22% of them were Muslim and Protestant followers, respectively. Regarding the head of the household, 221 (98.7%) of them were husbands. Concerning the maternal occupation, 153 (68.3%) of them were housewives (Table 1).

**TABLE 1:** Sociodemographic characteristics of study participants (*n* = 224), west Ethiopia, 2020.

Variable (category)	Frequency in number	Frequency (%)
Age group (in years)		
16–22	84	37.5
23–27	71	31.7
28–40	69	30.8
Marital status		
Divorced	7	3.1
Widowed	1	0.4
Maternal occupation		
Student	8	3.6
Daily laborer	19	8.5
Merchant	8	3.6
Governmental employee	3	1.3
Other	33	14.7

*Note*: Other: nongovernmental organizations (NGO).

### 3.2 Pregnancy and related characteristics of the study participants

Regarding the trimester of pregnancy, all of the respondents were in the first trimester and 37.1% of them had been two to three times pregnant. Concerning the interpregnancy interval, almost half (56.7%) of them had a 1‐year interval. Regarding iron/folate intake of pregnant women, 146 (65.2%) of them took iron/folate during their pregnancy (Table 2).

**TABLE 2:** Pregnancy and related characteristics of respondents (*n* = 224), west Ethiopia, 2020.

Variable (category)	Frequency in number	Frequency (%)
Number of pregnancies		
0–1	69	30.8
2–3	83	37.1
4–8	72	32.1
Interpregnancy interval (in years)		
0–1	127	56.7
2–5	97	43.3
Abortion		
Yes	20	8.9
No	204	91.1
ANC visits		
Yes	105	46.9
No	119	53.1

Abbreviation: ANC, antenatal care.

### 3.3 Dietary practice and related characteristics of respondents

The findings of this study showed that all the study participants (100%) have used Okra and 62.5% of them have daily consuming it. This study showed that 36.6%, 38.8%, and 83.5% of pregnant women used fruits, vegetables, and energy‐source food daily, respectively. In addition to this, 33% and 13.4% of the respondents changed their food intake and took extra food during pregnancy, respectively. More than one‐fifth (21%) of pregnant women avoided at least one food during their pregnancy. Similarly, 39.7%, 5.4%, and 0.4% of them skipped breakfast, lunch, and dinner respectively (Table 3).

**TABLE 3:** Dietary practice and related characteristics of respondents (*n* = 224), west Ethiopia, 2020.

Variable (category)	Frequency in number	Frequency (%)
Women's Dietary Diversity		
Low	129	57.6
Medium	31	13.8
High	64	28.6
Knowledge of diversified diet		
Poor	151	67.4
Good	73	32.6
Frequency of okra consumption		
Sometimes	84	37.5
Daily	140	62.5
Attitude toward diversified diet		
Negative	152	67.9
Positive	72	32.1

### 3.4 Nutritional status and related characteristics of respondents

The finding of this study revealed that 42.4% of pregnant women were malnourished (MUAC ≤ 22 cm). The results of chi‐square statistics indicated that there was a significant association at a 5% significance level between the nutritional status of pregnant women and the frequency of okra consumption (*χ* ^2^ = 42.61, df = 1, *p* < .001). For this reason, of the total pregnant women having low MUAC (undernourished), 62.1% of them have not used okra daily. In line with this, the results of chi‐squared statistics also indicated that there was a significant association between the nutritional status of pregnant women and the household food security status of pregnant women (*χ* ^2^ = 13.30, df = 1, *p* < .001). Accordingly, nearly half (47.4%) of malnourished women were food insecure (Table 4).

**FIGURE 1:**
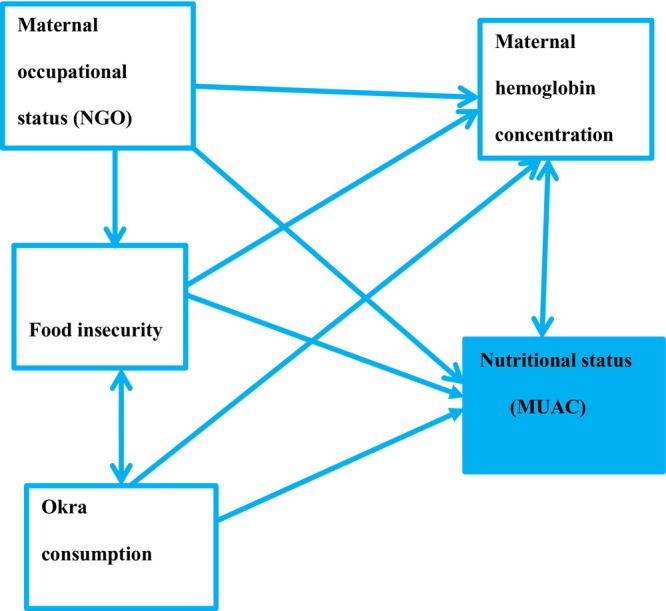
Graphical abstract for the impacts of okra consumption on the nutritional status of pregnant women, with correlations of okra consumption with different variables and the nutritional status of pregnant women. The prevalence of pregnant women with low mid‐upper arm circumference (MUAC) (≤22 cm) was 42.4%. Hemoglobin concentration, food insecurity, daily consumption of okra, and pregnant women working in NGOs were statistically significant variables that affect the nutritional status of pregnant women. Pearson correlation indicated that an increase in the frequency of okra consumption during pregnancy would lead to a higher MUAC measurement of pregnant women. Thus, the consumption of okra has a great impact on improving the nutritional status of pregnant women.

**TABLE 4:** Nutritional status and related characteristics of respondents (*n* = 224), west Ethiopia, 2020.

Variable (category)	Frequency in number	Frequency in percent (%)
Mid‐upper arm circumference		
≤22 cm	95	42.4
>22 cm	129	57.6
Hemoglobin concentration		
<11 g/dL	54	24.1
≥11 g/dL	170	75.9
Food insecurity status		
Secure	148	66.1
Insecure	76	33.9

### 3.5 Correlation analysis of variables

Pearson product correlation between the frequency of okra consumption in pregnancy and MUAC was found to be highly positive and statistically significant (*r* = .93, *p* < .001), which indicated that an increase in the frequency of okra consumption during pregnancy would lead to a higher MUAC measurement of pregnant women (Table 5 and Figure 1).

**TABLE 5:** Correlation analysis of variables for the impacts of okra consumption on the nutritional status of pregnant women, west Ethiopia, 2020.

	MUAC	Frequency of okra consumption	Hemoglobin concentration	Food insecurity	Occupation
MUAC	1				
Frequency of okra consumption	.93**	1			
Hemoglobin concentration	.47**	.85**	1		
Food insecurity	−.24**	−.21**	−.14*	1	
Occupation	.18**	−.20**	−.06	.19**	1

*Correlation is significant at the .05 level (two‐tailed); **Correlation is significant at the .01 level (two‐tailed).

### 3.6 Factors associated with maternal MUAC measurement

After adjusting for different confounding factors in the final multivariable linear regression model, the variables hemoglobin concentration (*β* = 0.346, [95% CI: 0.153, 0.539], *p* = .001), food insecurity (*β* = −0.887, [95% CI: −1.441, −0.334], *p* = .002), daily consumption of okra (*β* = 1.269, [95% CI: 0.583, 1.956], *p* ≤ .001), and women working in NGO (β = 0.443, [95% CI: 0.256, 0.630], *p* ≤ .001) were statistically significant at *p*‐value <.05 (Table 6 and Figure 1).

**TABLE 6:** Multivariable linear regression model predicting mid‐upper arm circumference measurement among pregnant women (*n* = 224), west Ethiopia, 2020.

Model	Unstandardized coefficients			95% CI		Multicollinearity test (VIF)
	*β*	Std. error	*p*‐Value	Lower	Upper	
Constant	19.790	2.260	.000	15.334	24.256	
Eating vegetables daily	0.482	0.280	.086	−0.070	1.035	1.470
ANC visits	0.483	0.296	.104	−0.101	1.068	1.464
Attitude	0.251	0.296	.396	−0.331	0.834	1.276
Hemoglobin concentration	0.334	0.098	.001*	0.140	0.528	1.898
Any meals skipped	−0.216	0.279	.439	−0.765	0.333	1.276
Food insecurity	−0.887	0.281	.002*	−1.441	−0.334	1.183
WDD	−0.178	0.384	.643	−0.936	0.580	2.686
Iron/folate intake	−0.040	0.317	.900	−0.666	0.586	1.531
Head of household	−0.583	1.302	.655	−3.149	1.983	1.499
Changing food intake	0.411	0.343	.233	−0.266	1.088	1.746
Frequency of okra consumption	1.296	0.352	.000**	0.602	1.991	1.945
Student dummy	−1.483	0.811	.069	−3.082	0.117	1.517
Housewife dummy	−0.511	0.415	.220	−1.328	0.307	2.491
Another occupation dummy	1.291	0.535	.017*	0.237	2.345	2.403

*Note*: *R* ^2^: .404.: Another occupation dummy: Non‐governmental organizations.

Abbreviations: *β* beta coefficient; WDD, Women Dietary Diversity.

*, Significance at *p*‐value <.05; **, Significance at *p*‐value ≤.001.

## 4 DISCUSSION

The findings of this study revealed that the mean (±SD) of MUAC among pregnant women was 22.65 (±2.28) cm. The prevalence of malnutrition among pregnant women (MUAC ≤ 22 cm) was 42.4%. This indicated that poor nutritional status (undernutrition) in pregnancy stated as a percent with low MUAC measurement was high in western Ethiopian women. This was slightly higher than that of a study conducted in three rural districts in the Oromia region of Ethiopia (41%) (Ghosh et al., 2019). Likewise, this finding was also higher than that of the study conducted among pregnant women in rural communities in southern Ethiopia, (41.2%) (Zewdie et al., 2021), but it was lower than that of the study conducted among pregnant women in the Kacha Birra District of southern Ethiopia (52.6%) (Teshome et al., 2021). This might be due to the difference in the study design, the study setting, and sociocultural factors that might affect their dietary practice. Those might affect the nutritional status (MUAC measurements) of women during pregnancy.

The finding of this study indicated that, for a unit (g/dL) increase in maternal hemoglobin concentration, maternal MUAC was increased by 0.346 cm. This indicated that there is a direct association between maternal hemoglobin concentration and MUAC measurements. Furthermore, the Pearson correlation between maternal hemoglobin concentration and MUAC measurements indicated there was moderately positive and statistically significant (*r* = .47, *p* < .001). This indicated that, as hemoglobin concentration increased, the size of maternal MUAC increased as well (Saaka et al., 2017a). There is also evidence that indicated that maternal MUAC was positively associated with maternal hemoglobin as well (Gebreegziabher et al., 2020). Furthermore, different evidence indicated that high bioavailability of iron was found in different parts of okra (Habtamu et al., 2018). In line with this, okra seed flour has different nutritional compositions: proteins, fat, and minerals such as iron and ant oxidative potentials, which are used for food fortification (Adelakun & Oyelade, 2011; Petropoulos et al., 2017; Zerihun et al., 2020). Moreover, okra leaves showed a predominance of minerals such as iron that was not significantly affected by food processing (Caluête et al., 2015). For this reason, frequent consumption of okra would help to prevent or reduce micronutrient deficiency problems along with the improvement of MUAC measurement of pregnant women in resource‐limited settings. Thus, mid‐upper arm circumference (MUAC) and hemoglobin concentration (anemia status) were used to assess the nutritional status of pregnant women.

According to the finding of this study, the MUAC measurement of food‐insecure pregnant women was decreased by 0.887 cm. This indicated that food insecurity was inversely associated with maternal MUAC measurements. Thus, food insecurity results in serious health problems in developing countries (Hasan et al., 2021; Motbainor et al., 2017). In line with this, food insecurity and poor‐quality diets remain a challenge for pregnant women (Maina et al., 2017). This indicated that food‐insecure households are more likely to be malnourished as a result of different coping strategies used which limit the quality and quantity of food intake. For this reason, the MUAC measurements of pregnant women might be decreased. Pregnant women from food‐insecure households in rural Bangladesh on average had 0.6 cm lower MUAC than their food‐secure counterparts (Hasan et al., 2021). On the contrary, food insecurity has been associated with poor pregnancy outcomes, including low birth weight and gestational diabetes (Borders et al., 2007; Laraia et al., 2010). However, evidence from rural areas of Northern Ghana indicated that food insecurity was not associated with the maternal thinness of pregnant women (Saaka et al., 2017b). Likewise, undernutrition during pregnancy is an important public health problem that is highly prevalent in Ethiopia (Gelebo et al., 2021). On the contrary, pregnant women in rural areas of the world who consumed a diversified diet had better dietary diversity scores (Shashikantha et al., 2016).

Enhancing sustainable smallholder productivity using indigenous and wild foods is an important international policy that is vital for achieving the 2030 Sustainable Development Goals (Leakey et al., 2021). In line with this, for a growing population, the sustainable way to eat a nutritionally balanced diet, save the land, and reduce greenhouse gas emissions is to consume and produce diets higher in plant‐based protein which will help to meet the Sustainable Development Goals (Kc et al., 2018). For this reason, the intake of a diverse diet consisting of locally available edible plants like okra is a cost‐effective strategy to overcome the food insecurity problems of pregnant women (Bhandari et al., 2016).

The finding of this study also indicated that daily consumption of okra was directly associated with MUAC measurement of pregnant women. Accordingly, on average those pregnant women who consume okra daily had 1.269 cm more MUAC measurement as compared to those who consumed it sometimes. This indicated that the consumption of wild edible plants like okra improves the nutritional status of pregnant women (Borelli et al., 2020; Carvalho & Barata, 2016; Grivetti & Ogle, 2000). In addition to this, wild edible plants contribute a critical role in ensuring food security and prevention of malnutrition particularly, for pregnant women (Salvi & Katewa, 2014). In line with this, okra is especially important for pregnant women due to its folate contents (Roy et al., 2014). Likewise, okra is a cost‐effective and economically affordable natural source that reduces malnutrition in resource‐limited settings (Elkhalifa et al., 2021). Similarly, the okra plant is a miracle plant that is a good source of minerals, vitamins, and nutrients that are important for pregnant women (Sindhu & Puri, 2016). Therefore, the use of such edible plants might be one input for the nutritional aspects of the Sustainable Development Goals (SDGs), which aimed to promote healthy and sustainable diets and ensure food security globally (Grosso et al., 2020).

According to the finding of this study, those pregnant women who worked in nongovernmental organizations (NGO) had on average 0.443 cm more MUAC measurement as compared to those who were housewives and students. This might be because those pregnant women who worked at NGOs might have a good quality of life, have more monthly income, and could afford a quality and diversified diet. In line with this, the quality of life of pregnant women is essential for improving maternal nutritional status and health during pregnancy (Estebsari et al., 2020). Evidence from Daegu, South Korea, indicated that job status affects the nutrient intake of pregnant women which affects the nutritional status of pregnant women (Jung & Choi, 2017). Likewise, the average household monthly income was significantly associated with the MUAC measurement of pregnant mothers (Tesfaye et al., 2022). Thus, those pregnant women who were working at NGO might have good monthly income and their MUAC was increased. The findings of this study imply that regular intake of okra has a crucial role in meeting the macro and micronutrient requirements of pregnant women, which in turn improves the nutritional status of pregnant women. It might also have policy implication that calls for further evidence that might have inputs that support efforts of sustainable development goals such as ending hunger, achieving food security, and improving nutrition among nutritionally vulnerable groups such as pregnant women.

## 5 CONCLUSION

The prevalence of malnutrition among pregnant women in western Ethiopia (MUAC ≤ 22 cm) was 42.4%. Hemoglobin concentration, food insecurity, daily consumption of okra, and pregnant women working in NGOs were statistically significant and associated variables that affect the nutritional status of pregnant women. Thus, the consumption of okra has a great impact on improving the nutritional status of pregnant women and also has a critical role in ensuring household food security as well. Therefore, dietary modification to ensure household food security, prevention of anemia, and behavioral change communication intervention to promote okra consumption on regular basis were recommended to increase the MUAC measurement of pregnant women. In line with this, strategies should aim to increase women's productivity and income (development of safety net programs), which maintains their household food security and nutrition. Moreover, public health measures should focus on ensuring proper nutrition during the critical growth periods of life (pregnancy) to prevent the adverse effects of household food insecurity (malnutrition) on women's health. Furthermore, increasing the agricultural productivity of okra was also recommended for food fortification, dietary diversification, and alleviation of problems associated with malnutrition in resource‐limited settings was very crucial as well.

## AUTHOR CONTRIBUTIONS

**Efrem Negash Kushi:** Conceptualization (lead); data curation (lead); formal analysis (equal); investigation (lead); methodology (equal); software (equal); supervision (equal); validation (supporting); writing – original draft (lead); writing – review and editing (equal). **Tefera Belachew:** Conceptualization (equal); formal analysis (equal); investigation (equal); methodology (equal); software (equal); supervision (equal); validation (equal); visualization (equal); writing – original draft (equal); writing – review and editing (equal). **Dessalegn Tamiru:** Conceptualization (equal); data curation (equal); formal analysis (equal); funding acquisition (equal); investigation (equal); methodology (equal); project administration (equal); resources (equal); software (equal); supervision (equal); validation (equal); visualization (equal); writing – original draft (equal); writing – review and editing (equal).

## FUNDING STATEMENT

This research received no specific grant from any funding agency in the public, commercial, or not‐for‐profit sectors.

## CONFLICT OF INTEREST STATEMENT

The authors declare that they do not have any conflict of interest.

## Data Availability

The datasets generated during and/or analyzed during the current study are available from the corresponding author upon reasonable request.
