# Correction to “The impacts of okra consumption on the nutritional status of pregnant women, west Ethiopia”

**DOI:** 10.1002/fsn3.3967

**Published:** 2024-01-10

Kushi, E. N., Belachew, T., & Tamiru, D. (2023). The impacts of okra consumption on the nutritional status of pregnant women, west Ethiopia. *Food Science & Nutrition*, 11, 5554–5564. https://doi.org/10.1002/fsn3.3512

In the originally published version of the article, an affiliation of Efrem Negash Kushi was missing: The correct authors and affiliations appear below.

Efrem Negash Kushi^1,2,*^, Tefera Belachew^1^, Dessalegn Tamiru^1^

^1^Department of Nutrition and Dietetics, Jimma University, Jimma, Ethiopia

^2^Department of Public Health, Mettu University, Mettu, Ethiopia

We apologize for this error.

